# Epidemiology of and risk factors for extrapulmonary nontuberculous mycobacterial infections in Northeast Thailand

**DOI:** 10.7717/peerj.5479

**Published:** 2018-08-16

**Authors:** Irin Kham-ngam, Ploenchan Chetchotisakd, Pimjai Ananta, Prajaub Chaimanee, Phuangphaka Sadee, Wipa Reechaipichitkul, Kiatichai Faksri

**Affiliations:** 1 Department of Microbiology, Faculty of Medicine, Khon Kaen University, Khon Kaen, Thailand; 2 Research and Diagnostic Center for Emerging Infectious Diseases, Khon Kaen University, Khon Kaen, Thailand; 3 Department of Medicine, Faculty of Medicine, Khon Kaen University, Khon Kaen, Thailand; 4 Clinical Laboratory Unit, Srinagarind Hospital, Faculty of Medicine, Khon Kaen University, Khon Kaen, Thailand

**Keywords:** Anti-IFN-γ, *Mycobacterium avium* complex, *Mycobacterium abscessus*, Nontuberculous mycobacteria, IFN-γ autoantibodies, Extrapulmonary NTM infection

## Abstract

**Background:**

Nontuberculous mycobacterial (NTM) infection is increasing worldwide. Current epidemiological data and knowledge of risk factors for this disease are limited. We investigated the trends in and risk of NTM infection in Northeast Thailand during 2012–2016.

**Methods:**

Patient demographics, infection site(s), and underlying disease or conditions from 530 suspected cases of NTM infections were retrieved from medical records, reviewed and analyzed. A diagnosis of true NTM infection was accepted in 150 cases. Risk factor analyses were done for extrapulmonary NTM infections compared to pulmonary NTM infections and for *Mycobacterium abscessus* compared to members of the *Mycobacterium avium* complex (MAC). Trend analysis among NTM species causing NTM infections was performed.

**Results:**

The most common species of NTMs causing extrapulmonary (*n* = 114) and pulmonary (*n* = 36) NTM infections in Northeast Thailand were *M. abscessus* (25.4% of extrapulmonary infected cases and 27.8% of pulmonary cases) followed by MAC (14.9% of extrapulmonary and 13.9% of pulmonary cases). Presence of anti-IFN-γ autoantibodies was the major risk factor for extrapulmonary (odds ratio (OR) = 20.75, 95%CI [2.70–159.24]) compared to pulmonary NTM infection. *M. abscessus* infection was less likely (OR = 0.17; 95%CI [0.04–0.80]) to be found in patients with HIV infection than was MAC infection. The prevalence of NTM infection, especially *M. abscessus*, in Northeast Thailand has recently increased. Extrapulmonary NTM and complicated NTM infections have increased in concordance with the recent trend of increasing frequency of anti-IFN-γ autoantibodies in the population.

**Conclusions:**

*M. abscessus* was the commonest NTM pathogen followed by MAC. The prevalence of NTM infections and anti-IFN-γ are showing an upward trend. Autoimmune disease due to anti-IFN-γ is the major risk factor for extrapulmonary NTM infection in Northeast Thailand.

## Introduction

Nontuberculous mycobacteria (NTMs) are aerobic, acid-fast bacilli belonging to the family Mycobacteriaceae. NTMs occur in the environment (Velayati et al., 2014) but some species can cause life-threatening disease in humans, with a high mortality (Cassidy et al., 2009; Iroh Tam et al., 2015). Although immunocompromised people are most at risk, NTMs can cause disease in immunocompetent individuals (Chetchotisakd et al., 2000; Wu & Holland, 2015). The incidence and prevalence of NTM disease are increasing worldwide (Ide et al., 2015; Shah et al., 2016), with different geographical patterns (Shao et al., 2015). In Asian countries, including Thailand, there is little epidemiological information concerning NTM disease.

Among NTM species, members of the *Mycobacterium avium* complex (MAC) and *Mycobacterium abscessus* are the most common in Asia (Chetchotisakd et al., 2007; Kim et al., 2015; Tang et al., 2015). *M. abscessus* infection mostly occurs as a chronic condition and is highly associated with drug resistance and treatment failure (Tung et al., 2015). MAC is the common NTM taxon causing NTM infection in humans (Griffith et al., 2007). This pathogen commonly causes disseminated NTM disease in HIV patients (Center for Disease Control, 2014). However, the risk analysis and current epidemiological status of these NTM diseases in Northeast Thailand has not yet been investigated.

Nontuberculous mycobacterial can caused both pulmonary and extrapulmonary infection such as lymph node infection, skin/soft tissue infection and disseminated infections. Previous studies have reported the risk factors for osteoarticular and skin infection caused by NTMs (Piersimoni & Scarparo, 2009). Very few studies have compared risk factors for pulmonary and extrapulmonary NTM infection (Umrao et al., 2016).

Several predisposing factors for NTM infections are known, such as older age and immune suppressive conditions (Cassidy et al., 2009). One of the most interesting risk factors for NTM infection is presence of anti-IFN-γ autoantibodies. This autoimmune condition is particularly found in Southeast Asia (Valour et al., 2016; Wongkulab et al., 2013) and has been found in NTM-infected patients without a diagnosis of clinical immunodeficiency (Aoki et al., 2018). Patients with NTM disease associated with anti-IFN-γ autoantibodies were almost always previously healthy and HIV-negative (Phoompoung et al., 2017). Although an association between anti-IFN-γ autoantibodies and NTM infection has been reported, it is not clear if there is any association between this autoimmune condition and complicated infection, as well as species of NTM.

We investigated the prevalence of, and risk factors for, NTM infection in Northeast Thailand during 2012–2016. Analysis was done of relative risks of pulmonary vs. extrapulmonary NTM infection and of *M. abscessus* vs. MAC infection. The frequency and trend of various NTM species causing NTM infections, from both pulmonary and extrapulmonary NTM infections were investigated. Demographic data, distribution of NTM infection across Northeast Thailand were analyzed.

## Methods

### Study population and setting

All patients received medical care at Srinagarind Hospital, a tertiary university hospital in Khon Kaen Province, Thailand. This is the largest hospital in Northeast Thailand, serving patients from several provinces there which have a combined population of around 20 million people. Clinical samples (*n* = 780) from 530 patients yielded NTMs in laboratory culture during the years 2012–2016. These constituted all consecutive NTM clinical samples and patients over that 5-year period. Each patient was suspected of mycobacterial infection and the samples sent for mycobacterial culture. Analysis of data from these patients forms the basis of this study. Cases were classified according to the criteria presented below. This study was approved by the Khon Kaen University Ethics Committee for Human Research (HE591454).

### Laboratory diagnosis

Specimens sent to the clinical laboratory were decontaminated using a standard NACL–NaOH method. Samples were inoculated into a liquid culture system (BACTEC™ MGIT™ 960; System BD, MD, USA). Discrimination between members of the *M. tuberculosis* complex and NTMs was done using the anti-MPT64 test (SD BIOLINE TB Ag MPT64 Rapid; SD, Gyeonggi-do, Korea). Cultures positive for NTMs were subcultured on Lowenstein–Jensen medium. NTM species identification was performed using INNO-LiPA MYCOBACTERIA v2 (INNOGENETICS GmbH, Heiden, Germany), Genotype *Mycobacterium* CM/AS assay (Hain Lifescience GmbH, Nehren, Germany) and Molecutech REBA Myco-ID (YD Diagnostics CORP., Gyeonggi-do, Korea). In some cases, species identity was confirmed by 16S rRNA gene sequencing (SolGent Co., Ltd., Seoul, Korea). Anti-HIV testing was performed according to the standard guidelines of Thailand (Bureau of AIDS TaS, 2017) and a positive result was noted when all three tests with different principles (P24 antigen detection, and anti-HIV detection and nucleic acid amplification testing) were positive. The anti-IFN-γ antibody test was routinely performed for many samples according to the ELISA technique described previously (Chetchotisakd et al., 2017) and the results noted in patients’ medical records. Patients for whom test results were not available were assumed to be negative in the analysis.

### Data collection

The clinical data (demographic data, underlying disease, organ involvement, laboratory test results and final diagnoses) of all patients were retrieved from medical records (Hospital Information System Database, Srinagarind Hospital, Khon Kaen, Thailand).

### Case definitions

True cases of NTM infection were defined as symptomatic patients receiving medical care with the isolation of NTM from sterile sites. Such sites include bone and joints, bone marrow, blood, eye, ascitic fluid, peritoneal dialysis samples, lymph node, pleural fluid, pleural tissue, bile duct, liver tissue, pericardial fluid, cerebrospinal fluid, neck tissue and other unspecified tissues. Due to the lack of radiological data, the case definition criteria according to ATS/IDSA guidelines (Griffith et al., 2007) could not be fully adopted. Isolation of NTMs from non-sterile sites/samples does not necessarily mean infection. Indeed, the majority of our samples were sputum (Table S1). When NTMs were isolated from non-sterile samples, criteria for defining a true case of NTM infection were as follows: exclusion of active tuberculosis; a medical record with a specific diagnosis of NTM infection made by the physician and clinical response to appropriate relevant antibiotics (i.e., clarithromycin, azithromycin, amikacin, cefoxitin, ciprofloxacin, doxycycline, ethambutol, isoniazid, imipenem, moxifloxacin, levofloxacin, ofloxacin, rifabutin, rifampicin, trimethoprim/sulfamethoxazole and tobramycin) (Center for Disease Control, 2014).

Pulmonary NTM infections were defined as true cases from which NTMs were isolated from pulmonary sites/samples such as sputum, pleural fluids, tissues, pus, lavage, suction, swab or washing fluids from nasal cavity, sinus tracts, trachea or pulmonary tissues. Extrapulmonary infection was defined based on isolation of NTMs from specimens outside the pulmonary sites listed above, or from both pulmonary and extrapulmonary sites. Simple NTM infection was defined as a true case due to a single NTM species in one organ. Mixed NTM infection refers to >1 species of NTM isolated from an individual patient. Multi-organ infections were defined as NTM infections occurring in >1 organ in an individual patient.

### Data analysis

Age and BMI are presented as mean ± SD values. Data for each NTM species causing true NTM infection are presented as percentage and proportion. Risk factor analysis for NTM infections in 104 extrapulmonary NTM cases, whose NTMs were isolated from sterile sites, and 46 pulmonary NTM cases (defined based on criteria above) was performed using chi-square and binary logistic regression and adjusted with statistically significant covariates. Odds ratios (ORs) were calculated to compare the risk of MAC vs. *M. abscessus* infection and pulmonary vs. extrapulmonary NTM infection. For the purposes of analysis, individuals with BMI >25 kg/m^2^ were regarded as overweight and individuals >50 years of age were regarded as belonging to an aging subpopulation. *p* < 0.05 was considered statistically significant. All statistical analyses were performed using SPSS version 17.0.

## Results

### Distribution of NTM isolated from clinical specimens and studied populations

**Figure 1:**
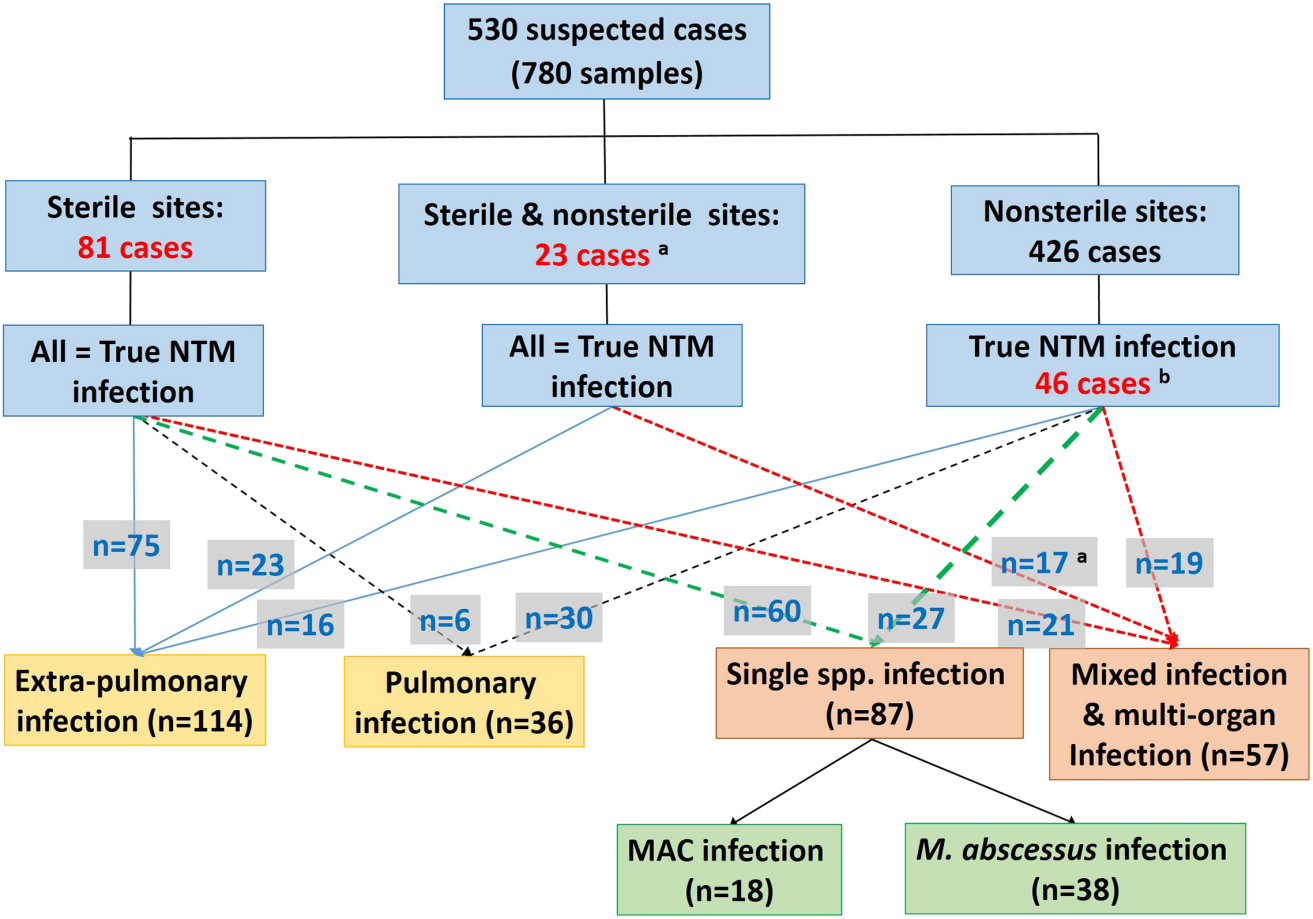
Studied populations and group classifications. ^a^NTMs were isolated from both sterile and nonsterile samples in six out of 23 cases, but no diagnosis and treatment data support the view that isolates from the nonsterile sites have contributed to infection. ^b^These cases were regarded as true NTM infections on the basis of their initial diagnoses and records of receiving relevant treatment (41 cases), or by only having a history of receiving relevant treatment (five cases).

The species distribution of NTMs isolated from clinical specimens of 530 suspected or proven patients (780 samples) is shown in Table S1. Of 530 suspected cases, 150 were defined as true NTM infections (Fig. 1). These 150 cases were further classified based on the criteria described above as (i) extrapulmonary (*n* = 114), (ii) pulmonary NTM infection (*n* = 36) and as simple (*n* = 87) and complicated (*n* = 57) NTM infections (Fig. 1). The most common species of NTM causing extrapulmonary and pulmonary NTM infections was *M. abscessus* (25.4% of extrapulmonary infected cases and 27.8% of pulmonary cases) followed by MAC (14.9% of extrapulmonary and 13.9% of pulmonary infected cases) (Table S2).

### Geographical distribution of NTM species causing NTM infections

**Figure 2:**
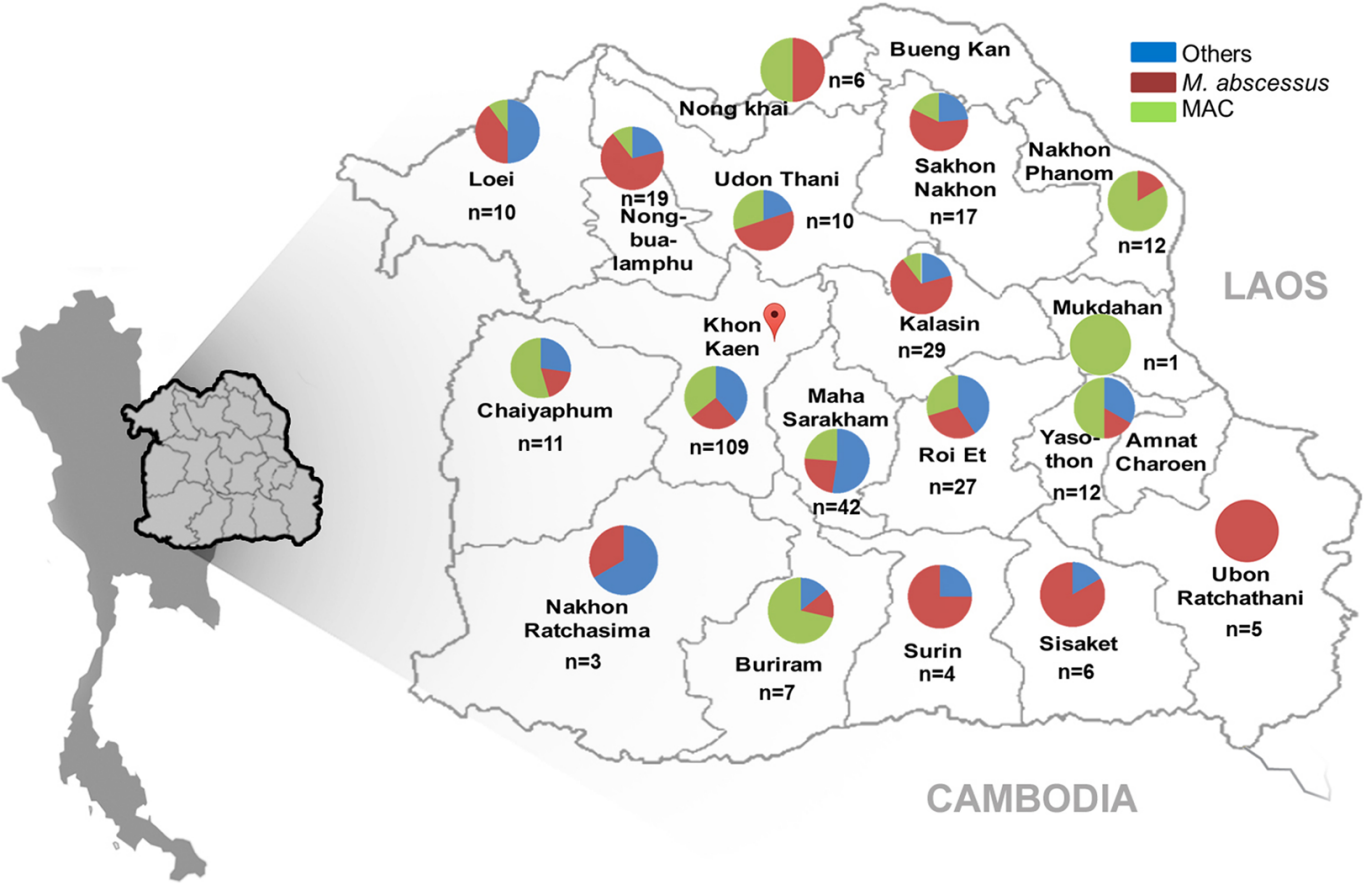
Geographical distribution of NTMs causing NTM infection Northeast Thailand. The distribution of NTM species (*n* = 330) isolated from 145 cases in Northeast Thailand is shown. Five NTM isolates came from patients outside Northeast Thailand. Map pin indicates the location of Srinagarind Hospital.

The geographical distribution of NTM species causing NTM infection in Northeast Thailand is shown in Fig. 2. Prevalence was highest in Khon Kaen Province. The proportions of MAC, *M. abscessus* and other NTMs causing NTM infection were roughly equally distributed among provinces in the center of Northeast Thailand (Fig. 2). In provinces on the periphery of the region, close to Laos, there was a predominance of MAC infections, whereas the southeast periphery of the region, close to Cambodia, had a predominance of *M. abscessus* cases (Fig. 2).

### Risk factors for extrapulmonary and pulmonary NTM infection

HIV (3/36 cases, 8.33%) and hypertension (3/36 cases) were the two most common underlying conditions in pulmonary NTM infections anti-IFN-γ autoantibodies were found in only one case (2.78%) from 36 pulmonary NTM infections (Table S3). The major underlying condition associated with extrapulmonary NTM infection was the presence of anti-IFN-γ autoantibodies (47.37%, 54/114 cases) (Table S4). Of the 54 cases of extrapulmonary NTM positive for anti-IFN-γ autoantibodies, 22 cases (40.74%) were mixed infections and 8 (14.81%) were multi-organ infections (Tables S4 and S5).

Risk factor analysis based on univariate analysis showed that patients with anti-IFN-γ autoantibodies (OR = 31.5, *p* < 0.0001), cutaneous lesions (*p* = 0.002) and aged younger than 50 years (*p* = 0.041) were more likely to suffer extrapulmonary NTM infection compared to pulmonary infection (Table 1). Multivariate analysis showed only anti-IFN-γ autoantibodies (OR = 20.75, *p* = 0.004) associated with extrapulmonary NTM infection.

**Table 1:** Risk factor analysis for extrapulmonary vs. pulmonary NTM infection.

Risk factors	Disease types		Crude analysis			Adjusted analysis		
	Pulmonary (*n* = 36)	Extra pulmonary (*n* = 114)	Crude OR	95% Cl	*p*-Value	Adjusted OR	95% Cl	*p*-Value
Age								
≥50	25 (69.44)	57 (50)	0.44	0.198–0.978	**0.041**	0.75	0.171–3.295	0.703
<50	11 (30.56)	57 (50)	1					
Gender								
Male	16 (44.44)	53 (46.49)	1.086	0.511–2.307	0.83	1.436	0.61–3.385	0.408
Female	20 (55.56)	61 (53.51)	1					
BMI^a^								
<25	25 (89.29)	66 (78.57)	0.44	0.119–1.624	0.208	0.286	0.067–1.217	0.09
≥25	3 (10.71)	18 (21.43)	1					
Anti-IFN-γ								
Yes	1 (2.78)	54 (47.37)	31.5	4.172–237.809	**<0.001**	20.749	2.704–159.235	**0.004**
No	35 (97.22)	62 (54.39)	1					
HIV								
Yes	3 (8.33)	14 (12.28)	1.54	0.417–5.694	0.764	1.59	0.37–6.837	0.533
No	33 (91.67)	100 (87.72)	1					
Cutaneous lesions^b^								
Yes	0 (0)	25 (21.93)	NA	NA	**0.002**	NA	NA	0.998
No	36 (100)	89 (78.07)	1					
Kidney disease								
Yes	2 (5.56)	21 (18.42)	3.839	0.854–17.249	0.062	4.729	0.973–22.987	0.054
No	34 (94.44)	93 (81.58)	1					
Cancer								
Yes	2 (5.56)	16 (14.04)	2.776	0.607–12.701	0.243	1.888	0.355–10.058	0.456
No	34 (94.44)	98 (85.96)	1					

**Notes:**

*p*-Value with bold fonts refer to statistically significant *p*-value.

a Some data were missing.

b Cutaneous lesions comprised of Sweet’s disease, eczema and erythema nodusum. Adjusted analysis was adjusted with the three most significant factors (anti-IFN-γ, cutaneous lesions and age). Odds ratios (ORs) compared extrapulmonary infections (numerator) to pulmonary infections (denominator).

### Risk factor analysis for *M. abscessus* infection and MAC infection

Risk factors for NTM infection (excluding mixed infection or multi-organ infection) caused by *M. abscessus* (38 cases) compared to MAC (18 cases) (the two most common NTM species isolated), were analyzed. Infection with *M. abscessus* was less likely (OR = 0.17; *p* = 0.024) to be found in patients with HIV infection than was MAC infection (Table 2).

**Table 2:** Risk factor analysis for *M. abscessus* vs. *M. avium* complex infection defined based on identities of NTMs isolated from sterile specimen sites.

Risk factors	Organisms		Crude analysis			Adjusted analysis		
	MAC (*n* = 18); *n* (%)	*M. abscessus* (*n* = 38); *n* (%)	Crude OR	95% Cl	*p*-Value	Adjusted OR	95% Cl	*p*-Value
Age								
≥50	8 (44.44)	26 (68.42)	2.708	0.854–8.59	0.086	1.66	0.446–6.186	0.45
<50	10 (55.56)	12 (31.58)	1					
Gender								
Male	12 (66.67)	19 (50)	0.5	0.155–1.608	0.241	0.625	0.183–2.14	0.455
Female	6 (33.33)	19 (50)	1					
BMI^a^								
<25	13 (86.67)	24 (88.89)	1.231	0.182–8.33	1.000	1.17	0.152–8.98	0.88
≥25	2 (13.33)	3 (11.11)	1					
Anti-IFN-γ								
Yes	4 (22.22)	13 (34.21)	1.82	0.497–6.662	0.362	1.7	0.436–6.625	0.444
No	14 (77.78)	25 (65.79)	1					
HIV positive								
Yes	6 (33.33)	3 (7.89)	0.171	0.037–0.794	**0.024**	0.171	0.037–0.794	**0.024**
No	12 (66.67)	35 (92.11)	1					
Cutaneous lesions^b^								
Yes	1 (5.56)	6 (15.79)	3.188	0.354–28.687	0.409	3.476	0.343–35.242	0.292
No	17 (94.44)	32 (84.21)	1					
Kidney disease								
Yes	1 (5.56)	7 (18.42)	3.839	0.435–33.863	0.414	2.75	0.302–25.026	0.369
No	17 (94.44)	31 (81.58)	1					
Cancer								
Yes	1 (5.56)	6 (15.79)	3.839	0.435–33.863	0.414	3.476	0.343–35.242	0.292
No	17 (94.44)	32 (84.21)	1					

**Notes:**

*p*-Value with bold fonts refer to statistically significant *p*-value.

a Some data were missing.

b Cutaneous lesions included Sweet’s disease, eczema and erythema nodusum. Adjusted analysis was adjusted with HIV status. Odds ratios (ORs) compared *M. abscessus* infection (numerator) vs. MAC infection (denominator). To exclude the confounding factors from mixed infections and multi-organ infections, only single-species infections caused by MAC or *M. abscessus* were included in the analysis. MAC, *Mycobacterium avium* complex.

### Prevalence and trends in NTMs infection during 2012–2016

**Figure 3:**
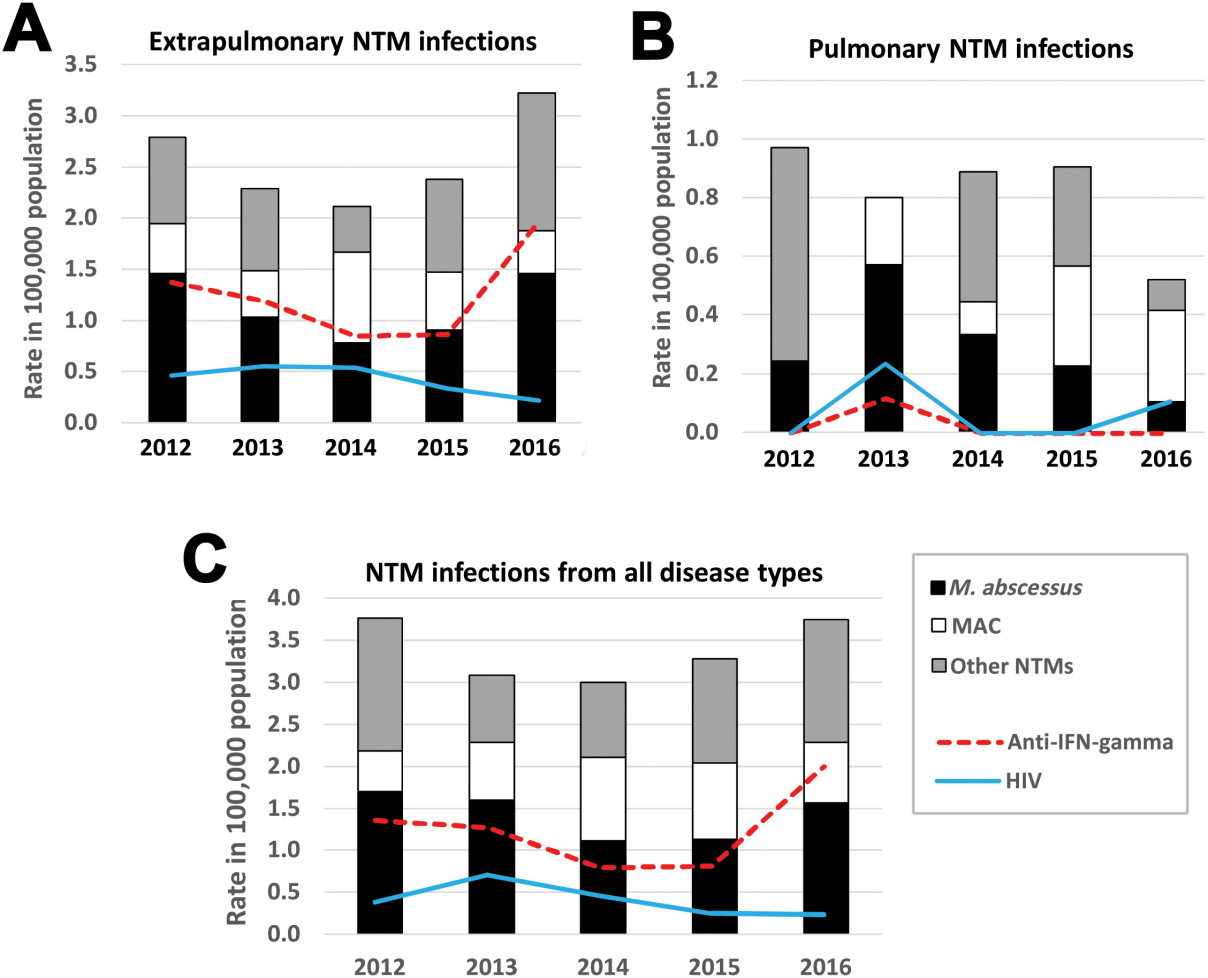
The 5-year trend in NTM infection cases between 2012 and 2016 in Northeast Thailand. The 5-year trend in the prevalence (per 100,000 hospital patient population) of extrapulmonary NTM infection (A), pulmonary NTM infection (B) and all types of NTM infection (C). All true NTM infections (*n* = 150) are plotted proportional to the number of patients in Srinagarind Hospital in the relevant year. The prevalence of anti-IFN-γ autoantibodies and HIV infection occurring in NTM infected cases are also shown. The total numbers of patients (including outpatients) visiting Srinagarind Hospital in the years 2012–2016 were 823,571, 874,195, 899,747, 883,075 and 960,664 cases, respectively.

Changes in prevalence of NTM infections from 2012 until 2016 is shown in Fig. 3. In the first 3 years, the prevalence of NTM cases and anti-IFN-γ autoantibodies from extrapulmonary NTM infection (Fig. 3A) and all NTM infections (Fig. 3C) decreased but then increased in the year 2016. There was no trend of increasing pulmonary NTM infection (Fig. 3B). The incidence of HIV found in NTM cases decreased but prevalence of anti-IFN-γ autoantibodies increased (Fig. 3C).

## Discussion

The prevalences of NTM species causing human disease vary depending on the geographical region (Ide et al., 2015; Shao et al., 2015). An association between the environmental NTMs from each region and NTM disease has been reported (Velayati et al., 2014, 2015). Generally, members of the MAC have been the most common NTMs found worldwide, especially in pulmonary specimens of suspected NTM-infected cases (Hoefsloot et al., 2013). In Western countries, such as England (Shah et al., 2016) and the US (Smith et al., 2016), MAC was the most common group of NTMs detected. However, *M. abscessus* was the most commonly found species in Scandinavia (Qvist et al., 2015). In Asian countries, MAC is also the most common taxon isolated from respiratory specimens (Simons et al., 2011). However, there is regional variation in the commonest types of NTM. For example, *Mycobacterium intracellulare* was the most common pathogen in Eastern China (Shao et al., 2015) and Southern Japan (Ide et al., 2015). In Korea, MAC and *M. abscessus* were the most common NTM taxa isolated from respiratory specimens (Jang et al., 2014). In our study, MAC and *M. abscessus* are the most prevalent taxa of NTMs isolated from all clinical specimens and caused disease in both intra- and extrapulmonary sites. The most common species was *M. abscessus*, which mostly caused lymphadenopathy. This was followed by MAC, members of which caused disseminated infections in blood, bone marrow, joints and bone. This result indicated that prevalence of NTM species was also associated with particular organ systems. *M. abscessus* is the most important species causing NTM infection in Northeast Thailand.

As the geographical distribution of NTM infection varies by region (Hoefsloot et al., 2013), we analyzed the distribution of NTMs isolated from specimens in Northeast Thailand. Khon Kaen Province had the highest prevalence of NTM infections. However, Srinagarind Hospital is located in this province, which might be a confounding factor. The proportions of NTM species found from each province near the center of Northeast Thailand were roughly comparable, that is, MAC and *M. abscessus* were the most prevalent species. Sample sizes were small from some peripheral provinces, but the results suggest a predominance of the MAC in the periphery close to Laos and *M. abscessus* in the region close to Cambodia and might suggest an association of predominance of NTM species and geographical region.

The diagnostic criteria for lung diseases caused by NTM should be based on clinical, radiological and microbiological criteria, and there needs to be an emphasis on the exclusion of other lung diseases, especially pulmonary tuberculosis (Center for Disease Control, 2014). We applied such criteria as far as possible, except for radiological data, that could not be fully accessed. Alternatively, we defined possible true infections of NTM when these organisms were isolated from non-sterile specimens (sputum, bronchial wash and bronchial lavage) of symptomatic patients who had a record of related treatment and/or specific diagnosis. In most such cases, a specific diagnosis had been made and relevant treatment, in addition to microbiological evidence, had been recorded. The commonest species causing both pulmonary and extrapulmonary NTM infections were *M. abscessus* and MAC. However, we could not be certain of detecting all cases of lung disease caused by NTM.

Presence of anti-IFN-γ autoantibodies is the single most important specific condition predisposing to NTM infection. Previously, these autoantibodies were detected in 81% of 52 disseminated NTM infection cases (Browne et al., 2012). In another study, anti-IFN-γ autoantibodies were found in six out of 35 patients with disseminated NTM infection and their presence was suggested as a risk factor for NTM infection associated with autoimmune disease and immunodeficiency (Patel et al., 2005). There is also a recent literature review of 64 cases of anti-IFN-γ autoantibodies associated with NTM infection (Valour et al., 2016). In Thailand, presence of anti-IFN-γ autoantibodies is associated with disseminated NTM disease and with generalized lymphadenitis and reactive skin lesions (Phoompoung et al., 2017). Anti-IFN-γ autoantibodies were reported as a risk factor for NTM infection in Thailand and Taiwan (Browne et al., 2012). We also analyzed the association between NTM infection and underlying diseases/conditions relating to immunodeficiency. The risk factors for pulmonary and extrapulmonary NTM infection were analyzed. We found that presence of anti-IFN-γ autoantibodies was strongly associated with extrapulmonary NTM infection and the risk was increased more than 20-fold relative to the risk of pulmonary NTM infection. Furthermore, more than half of the NTM infections found in anti-IFN-γ autoantibodies-positive individuals were either mixed infections or multi-organ infections. This might reflect the role of IFN-γ in the immune system of the host to control NTM infection outside the lung and disseminated NTM infection. Anti-IFN-γ autoantibodies have been found in the majority of the extrapulmonary NTM infections (Chi et al., 2013; Koya et al., 2009). However, no previous study has showed the risk factor analysis relative to pulmonary NTM infection (as reported here). In one study comparing risk factors for pulmonary and extrapulmonary NTM infection (Umrao et al., 2016), investigation of anti-IFN-γ autoantibodies was not done.

We found a trend of increasing incidence of anti-IFN-γ autoantibodies in Northeast Thailand. However, full investigation of all patients might reveal our observed prevalences of anti-IFN-γ autoantibodies from extrapulmonary (47.4%) and pulmonary (2.8%) NTM infections to be underestimates. Our information about presence of anti-IFN-γ autoantibodies and about HIV status came from the medical records of each patient. For some patients, no relevant tests had been done. This is a limitation of our study. We have treated these cases as negative results in the analysis due to the absence of associated clinical symptoms, such as reactive skin lesions and opportunistic infections such as disseminated salmonellosis. HIV testing was only done on patients with relevant symptoms and/or a history of risk factors: the assumption of a negative result in those not tested is therefore likely to be correct. Taken together, these results reinforce the view that presence of anti-IFN-γ autoantibodies is the most important risk factor for NTM infection.

There have been few studies reporting trends in NTM prevalence. These generally report an increasing trend, that is, China (2004–2014) (Velayati et al., 2014), (2008–2012) (Wu et al., 2014) and Taiwan (2002–2007) (Tsai et al., 2011). In our study, the total numbers of NTMs infections, especially extrapulmonary NTM infections, showed a decreasing trend in the first three years (2012–2014), but increased in 2015–2016. The trend of increasing incidence of anti-IFN-γ autoantibodies might explain the increase in NTM infections in Northeast Thailand. The decreasing trend of NTM infection in 2012–2014 might be a response to use of highly active antiretroviral therapy (HAART) in HIV-infected patients in Thailand (Bureau of AIDS TaS, 2017). Previously, HIV infection was the major immunodeficiency disease associated with NTM infection (Henkle & Winthrop, 2015; McCarthy et al., 2012), but we found this to be no longer the case in Thailand: only 17 HIV cases were among our 150 NTM infected patients. Thailand has effective national guidelines of HAART for HIV-infected persons that can improve the level of immunity of the patients (Bureau of AIDS TaS, 2017), perhaps leading to a decrease of the prevalence of NTM infection in HIV-infected persons. However, our result suggested that HIV infection was still a risk factor for MAC infection compare to *M. abscessus* infection.

The limitations of the epidemiological data from our study should be noted. The studied population in our study was from a single hospital. However, Srinagarind Hospital is a super-tertiary hospital serving all provinces in Northeast Thailand. Not all standard criteria for the diagnosis of lung diseases caused by NTMs could be applied due to the lack of radiological data. Due to the limitation of species identification tests, some NTMs were identified only as *Mycobacterium* sp. or as “rapid growers”. We defined most of true NTM infection by the isolation of NTMs from sterile clinical specimens of symptomatic persons. Most of the sterile specimens in our study were from deep organs (except ocular samples) and likely indicated disseminated NTM infections.

## Conclusion

The most common NTMs causing extrapulmonary and pulmonary NTM infections in Northeast Thailand were *M. abscessus* and the MAC. Presence of anti-IFN-γ autoantibodies was the major risk factor for extrapulmonary NTM infection. Individuals with anti-IFN-γ autoantibodies tended to have mixed NTM infections or multi-organ infections. The prevalence of NTM infection, especially infection due to *M. abscessus*, has recently increased in Northeast Thailand. Extrapulmonary NTM has recently increased in concordance with the recent trend of increased prevalence of anti-IFN-γ autoantibodies. Compared with *M. abscessus* infection, HIV remains a significant risk factor for MAC infection. This study provides current overall epidemiological data of NTM infection in Thailand.

## Supplemental Information

10.7717/peerj.5479/supp-1 Supplemental Information 1 Table S1. Overall distribution of NTM species isolated from clinical specimens stratified by specimen collection sites. Others (pulmonary site) refers to pleural tissue (1 case), pus from sinus tracts (2 cases), trachea tissue (1 case), pus from nasal cavity (1 case) and swab nasal cavity (1 case). Others (non-pulmonary sites) referred to bile duct (1 case), cerebrospinal fluid (1 case), liver tissue (1 case), neck tissue (1 case), pericardium fluid (3 cases) unspecified abscess (16 cases), unspecified fluid (5 cases) and unidentified samples (11 cases). GI refers to gastrointestinal tract comprised of stool (4 cases), ascitic fluid (1 case), gastric content (1 case) and peritoneal dialysis (2 cases). The total number of NTM isolates (*n* = 780) did not count the number of the same species isolated from serially collected specimens. MAC = *Mycobacterium avium* complex.

10.7717/peerj.5479/supp-2 Supplemental Information 2 Table S2. Distribution of NTM Species causing NTM infection stratified by site of infections (335 isolates from 150 cases). **Note:** NTMs were isolated from 97 pulmonary samples (36 cases) and 238 extra-pulmonary samples (114 cases). Mixed NTM refers to isolation of >1 species of NTM from the specimens, i.e. from pulmonary samples includes *M. intracellulare* and *M. avium* (1 case) and *M. massiliense* and *M. abscessus* (1 case), and from extra-pulmonary samples includes MAC and *M. intracellulare* (1 case), *M. gordonae* and *M. simiae* (2 cases), *M. fortuitum* and *M. abscessus* (1 case), *M. fortuitum* and *M. peregrinum* (3 cases) and *M. intracellulare* and *M. scrofulaceum* (1 case). MAC = *Mycobacterium avium* complex.

10.7717/peerj.5479/supp-3 Supplemental Information 3 Table S3. Risk factors of pulmonary NTM infection (36 cases).

10.7717/peerj.5479/supp-4 Supplemental Information 4 Table S4. Risk factors for extrapulmonary NTM infection (114 cases). NTMs were isolated from 114 patients with extra-pulmonary infections. Mixed infection (32 cases) refers to >1 species of NTM isolated from the same specimen or multiple specimen types from a single individual. Multi-organ infection (11 cases) refers to isolation of the same NTM species from various organ sites from an individual patient. Missing data (no test result or record specified in the medical records) for BMI (*n* = 30 cases), anti–IFN-γ (*n* = 59 case) and HIV (*n* = 38 cases). Patients for whom tests for HIV and /or anti–IFN-γ autoantibodies were not done had presented none of the associated symptoms and/or, in the case of HIV, had no risk factors reported in their histories. We have treated these cases as negative results in the analysis. “Others” referred to cholestatic hepatitis, histoplasmosis, arthritis, cryptococcosis, fungal keratitis, liver disease, osteonecrosis, thalassaemia, shigellosis, melioidosis, myelodysplastic syndromes, pneumonia, cerebrovascular disease, Kaposi’s sarcoma, bronchitis, Parkinson’s disease, parasitic diseases, plane wart, Salmonella septicaemia, lymphadenopathy, necrotising fasciitis and Kimura’s disease. MAC = *Mycobacterium avium* complex, RGM = Rapid growing Mycobacteria.

10.7717/peerj.5479/supp-5 Supplemental Information 5 Table S5. Details of mixed infections and multi-organ NTM infections (total = 57 cases). Mixed infection refers to >1 species of NTM isolated from the sample specimen or multiple specimens from the same NTM-infected case. Multi-organ infection refers to single NTM species isolated from multiple organ sites from an individual patient. From the total of 57 cases, 41 were defined based on NTM isolation from sterile sites only. In 16 cases, NTMs were isolated from both sterile and non-sterile specimens including 10 cases of mixed infection and 6 cases of multiple-organ infection. MAC = *Mycobacterium avium* complex.

10.7717/peerj.5479/supp-6 Supplemental Information 6 Raw data.

## Additional Information and Declarations

### Data Availability

The following information was supplied regarding data availability: The raw data are provided in the Supplemental Files.
