# Supplementary material for: Epidemiology of and risk factors for extrapulmonary nontuberculous mycobacterial infections in Northeast Thailand
Source: PeerJ. 2018 Aug 16;6:e5479. doi: 10.7717/peerj.5479 (PMC6098943; doi:10.7717/peerj.5479)
Supplement: Supplemental Information 1 — Others (pulmonary site) refers to pleural tissue (1 case), pus from sinus tracts (2 cases), trachea tissue (1 case), pus from nasal cavity (1 case) and swab nasal cavity (1 case). Others (non-pulmonary sites) referred to bile duct (1 case), cerebrospinal fluid (1 case), liver tissue (1 case), neck tissue (1 case), pericardium fluid (3 cases) unspecified abscess (16 cases), unspecified fluid (5 cases) and unidentified samples (11 cases). GI refers to gastrointestinal tract comprised of stool (4 cases), ascitic fluid (1 case), gastric content (1 case) and peritoneal dialysis (2 cases). The total number of NTM isolates (n = 780) did not count the number of the same species isolated from serially collected specimens. MAC = Mycobacterium avium complex. [file peerj-06-5479-s001.docx]

**SUPPLEMENTAL INFORMATION**

**Table S1** Overall distribution of NTM species isolated from clinical specimens stratified by specimen collection sites.

| **Species** | **Bone&joint n (%)** | **Blood&Bone Marrow n (%)** | **Cutaneous**  **n (%)** | **Eye**  **n (%)** | **GI**  **n (%)** | **Lymph node**  **n (%)** | **Pulmonary** | | | | **Unspecified Tissue n (%)** | **Urine n (%)** | **Others**  **n (%)** | **Total**  **n (%)** |
| --- | --- | --- | --- | --- | --- | --- | --- | --- | --- | --- | --- | --- | --- | --- |
|  |  |  |  |  |  |  | **Sputum n (%)** | **lavage/wash/**  **suction n (%)** | **Pleural fluid n (%)** | **Others n (%)** |  |  |  |  |
| *M. abscessus* | 0 (0) | 6 (15.38) | 9 (20.45) | 6 (75) | 2 (25) | 36 (59.02) | 90 (19.27) | 13 (20.97) | 4 (50) | 1 (16.67) | 7 (50) | 1 (20) | 13 (33.33) | 188 (24.1) |
| *M. arupense* | 0 (0) | 0 (0) | 0 (0) | 0 (0) | 0 (0) | 0 (0) | 1 (0.21) | 0 (0) | 0 (0) | 0 (0) | 0 (0) | 0 (0) | 0 (0) | 1 (0.13) |
| *M. asiaticum* | 0 (0) | 0 (0) | 1 (2.27) | 0 (0) | 0 (0) | 1 (1.64) | 2 (0.43) | 1 (1.61) | 0 (0) | 0 (0) | 0 (0) | 0 (0) | 1 (2.56) | 6 (0.77) |
| MAC (all species) |  |  |  |  |  |  |  |  |  |  |  |  |  |  |
| *M. avium* | 0 (0) | 2 (5.13) | 0 (0) | 0 (0) | 0 (0) | 0 (0) | 1 (0.21) | 0 (0) | 0 (0) | 0 (0) | 0 (0) | 0 (0) | 1 (2.56) | 4 (0.51) |
| *M. intracellulare* | 12 (63.16) | 4 (10.26) | 9 (20.45) | 0 (0) | 0 (0) | 1 (1.64) | 95 (20.34) | 16 (25.81) | 0 (0) | 1 (16.67) | 3 (21.43) | 0 (0) | 6 (15.38) | 147 (18.85) |
| Unidentified MAC | 4 (21.05) | 5 (12.82) | 9 (20.45) | 0 (0) | 1 (12.5) | 2 (3.28) | 52 (11.13) | 5 (8.06) | 0 (0) | 0 (0) | 0 (0) | 0 (0) | 2 (5.13) | 80 (10.26) |
| *M. chelonae* | 0 (0) | 1 (2.56) | 1 (2.27) | 0 (0) | 0 (0) | 3 (4.92) | 7 (1.5) | 0 (0) | 1 (12.5) | 0 (0) | 0 (0) | 0 (0) | 0 (0) | 13 (1.67) |
| *M. florentinum* | 0 (0) | 0 (0) | 0 (0) | 0 (0) | 0 (0) | 0 (0) | 1 (0.21) | 0 (0) | 0 (0) | 0 (0) | 0 (0) | 0 (0) | 0 (0) | 1 (0.13) |
| *M. fortuitum* | 0 (0) | 0 (0) | 2 (4.55) | 0 (0) | 1 (12.5) | 0 (0) | 30 (6.42) | 2 (3.23) | 1 (12.5) | 2 (33.33) | 1 (7.14) | 1 (20) | 1 (2.56) | 41 (5.26) |
| *M. genavense* | 0 (0) | 0 (0) | 0 (0) | 0 (0) | 0 (0) | 0 (0) | 6 (1.28) | 0 (0) | 0 (0) | 0 (0) | 0 (0) | 0 (0) | 1 (2.56) | 7 (0.9) |
| *M. gordonae* | 0 (0) | 0 (0) | 1 (2.27) | 0 (0) | 0 (0) | 0 (0) | 19 (4.07) | 0 (0) | 0 (0) | 0 (0) | 0 (0) | 0 (0) | 0 (0) | 20 (2.56) |
| *M. interjectum* | 0 (0) | 0 (0) | 0 (0) | 0 (0) | 0 (0) | 0 (0) | 4 (0.86) | 0 (0) | 0 (0) | 0 (0) | 0 (0) | 0 (0) | 0 (0) | 4 (0.51) |
| *M. kansasii* | 1 (5.26) | 1 (2.56) | 0 (0) | 0 (0) | 0 (0) | 2 (3.28) | 6 (1.28) | 2 (3.23) | 1 (12.5) | 0 (0) | 0 (0) | 0 (0) | 0 (0) | 13 (1.67) |
| *M. lentiflavum* | 0 (0) | 0 (0) | 0 (0) | 0 (0) | 0 (0) | 0 (0) | 7 (1.5) | 0 (0) | 0 (0) | 0 (0) | 0 (0) | 0 (0) | 0 (0) | 7 (0.9) |
| *M. malmoense* | 0 (0) | 0 (0) | 0 (0) | 0 (0) | 0 (0) | 0 (0) | 2 (0.43) | 1 (1.61) | 0 (0) | 0 (0) | 0 (0) | 0 (0) | 0 (0) | 3 (0.38) |
| *M. marinum* | 0 (0) | 0 (0) | 0 (0) | 0 (0) | 0 (0) | 1 (1.64) | 1 (0.21) | 0 (0) | 0 (0) | 0 (0) | 0 (0) | 0 (0) | 0 (0) | 2 (0.26) |
| *M. monacense* | 0 (0) | 0 (0) | 0 (0) | 0 (0) | 0 (0) | 0 (0) | 1 (0.21) | 0 (0) | 0 (0) | 0 (0) | 0 (0) | 0 (0) | 0 (0) | 1 (0.13) |
| *M. mucogenicum* | 0 (0) | 0 (0) | 0 (0) | 0 (0) | 0 (0) | 0 (0) | 2 (0.43) | 0 (0) | 0 (0) | 0 (0) | 0 (0) | 0 (0) | 0 (0) | 2 (0.26) |
| *M. palustre* | 0 (0) | 0 (0) | 0 (0) | 0 (0) | 0 (0) | 0 (0) | 3 (0.64) | 0 (0) | 0 (0) | 0 (0) | 0 (0) | 0 (0) | 0 (0) | 3 (0.38) |
| *M. saskatchewanense* | 0 (0) | 0 (0) | 0 (0) | 0 (0) | 0 (0) | 0 (0) | 0 (0) | 1 (1.61) | 0 (0) | 0 (0) | 0 (0) | 0 (0) | 0 (0) | 1 (0.13) |
| *M. scrofulaceum* | 1 (5.26) | 0 (0) | 3 (6.82) | 0 (0) | 1 (12.5) | 1 (1.64) | 22 (4.71) | 1 (1.61) | 0 (0) | 0 (0) | 2 (14.29) | 0 (0) | 1 (2.56) | 32 (4.1) |
| *M. simiae* | 0 (0) | 0 (0) | 1 (2.27) | 0 (0) | 0 (0) | 0 (0) | 4 (0.86) | 2 (3.23) | 0 (0) | 0 (0) | 0 (0) | 0 (0) | 0 (0) | 7 (0.9) |
| *M. szulgai* | 1 (5.26) | 0 (0) | 0 (0) | 0 (0) | 0 (0) | 0 (0) | 3 (0.64) | 1 (1.61) | 0 (0) | 0 (0) | 0 (0) | 0 (0) | 0 (0) | 5 (0.64) |
| *Mycobacterium* spp. | 0 (0) | 1 (2.56) | 3 (6.82) | 2 (25) | 1 (12.5) | 7 (11.48) | 80 (17.13) | 12 (19.35) | 0 (0) | 1 (16.67) | 0 (0) | 2 (40) | 8 (20.51) | 117 (15) |
| Rapid grower | 0 (0) | 13 (33.33) | 4 (9.09) | 0 (0) | 2 (25) | 7 (11.48) | 11 (2.36) | 1 (1.61) | 0 (0) | 0 (0) | 1 (7.14) | 1 (20) | 3 (7.69) | 43 (5.51) |
| Mixed NTM | 0 (0) | 6 (15.38) | 1 (2.27) | 0 (0) | 0 (0) | 0 (0) | 17 (3.64) | 4 (6.45) | 1 (12.5) | 1 (16.67) | 0 (0) | 0 (0) | 2 (5.13) | 32 (4.1) |
| **Total** | **19 (100)** | **39 (100)** | **44 (100)** | **8 (100)** | **8 (100)** | **61 (100)** | **467 (100)** | **62 (100)** | **8 (100)** | **6 (100)** | **14 (100)** | **5 (100)** | **39 (100)** | **780 (100)** |

Others (pulmonary site) refers to pleural tissue (1 case), pus from sinus tracts (2 cases), trachea tissue (1 case), pus from nasal cavity (1 case) and swab nasal cavity (1 case). Others (non-pulmonary sites) referred to bile duct (1 case), cerebrospinal fluid (1 case), liver tissue (1 case), neck tissue (1 case), pericardium fluid (3 cases) unspecified abscess (16 cases), unspecified fluid (5 cases) and unidentified samples (11 cases). GI refers to gastrointestinal tract comprised of stool (4 cases), ascitic fluid (1 case), gastric content (1 case) and peritoneal dialysis (2 cases). The total number of NTM isolates (n=780) did not count the number of the same species isolated from serially collected specimens. MAC=*Mycobacterium avium* complex.
