# Supplementary material for: Epidemiology of and risk factors for extrapulmonary nontuberculous mycobacterial infections in Northeast Thailand
Source: PeerJ. 2018 Aug 16;6:e5479. doi: 10.7717/peerj.5479 (PMC6098943; doi:10.7717/peerj.5479)
Supplement: Supplemental Information 2 — Note: NTMs were isolated from 97 pulmonary samples (36 cases) and 238 extra-pulmonary samples (114 cases). Mixed NTM refers to isolation of >1 species of NTM from the specimens, i.e. from pulmonary samples includes M. intracellulare and M. avium (1 case) and M. massiliense and M. abscessus (1 case), and from extra-pulmonary samples includes MAC and M. intracellulare (1 case), M. gordonae and M. simiae (2 cases), M. fortuitum and M. abscessus (1 case), M. fortuitum and M. peregrinum (3 cases) and M. intracellulare and M. scrofulaceum (1 case). MAC = Mycobacterium avium complex. [file peerj-06-5479-s002.docx]

**Table S2** Distribution of NTM Species causing NTM infection stratified by site of infections (335 isolates from 150 cases).

| **Organisms** | **Number of isolates (335 isolates):**  **n (%)** | | |  | **Number of cases (150 cases):**  **n (%)** | | |
| --- | --- | --- | --- | --- | --- | --- | --- |
|  | **Pulmonary** | **Extra pulmonary** | **Total** |  | **Pulmonary** | **Extra pulmonary** | **Total** |
| *M. abscessus* | 34 (35.05) | 88 (36.97) | 122 (36.42) |  | 10 (27.78) | 29 (25.44) | 39 (26) |
| *M. arupense* | 0 (0) | 0 (0) | 0 (0) |  | 0 | 0 | 0 (0) |
| *M. asiaticum* | 0 (0) | 2 (0.84) | 2 (0.6) |  | 0 | 1 (0.88) | 1 (0.67) |
| MAC (all species) | *30 (30.93)* | *69 (28.99)* | *99 (29.56)* |  | *5 (13.89)* | *17 (14.91)* | *22 (14.67)* |
| *M. avium* | 0 (0) | 3 (1.26) | 3 (0.9) |  | 0 | 3 (2.63) | 3 (2) |
| *M. intracellulare* | 22 (22.68) | 38 (15.97) | 60 (17.91) |  | 3 (8.33) | 7 (6.14) | 10 (6.67) |
| Unidentified MAC | 8 (8.25) | 28 (11.76) | 36 (10.75) |  | 2 (5.56) | 7 (6.14) | 9 (6) |
| *M. chelonae* | 3 (3.09) | 7 (2.94) | 10 (2.99) |  | 1 (2.78) | 3 (2.63) | 4 (2.67) |
| *M. florentinum* | 0 (0) | 0 (0) | 0 (0) |  | 0 | 0 | 0 (0) |
| *M. fortuitum* | 6 (6.19) | 5 (2.1) | 11 (3.28) |  | 3 (8.33) | 2 (1.75) | 5 (3.33) |
| *M. genavense* | 0 (0) | 1 (0.42) | 1 (0.3) |  | 0 | 1 (0.88) | 1 (0.67) |
| *M. gordonae* | 4 (4.12) | 0 (0) | 4 (1.19) |  | 1 (2.78) | 0 | 1 (0.67) |
| *M. interjectum* | 0 (0) | 0 (0) | 0 (0) |  | 0 | 0 | 0 (0) |
| *M. kansasii* | 1 (1.03) | 6 (2.52) | 7 (2.09) |  | 0 | 0 | 0 (0) |
| *M. lentiflavum* | 0 (0) | 0 (0) | 0 (0) |  | 0 | 0 | 0 (0) |
| *M. malmoense* | 1 (1.03) | 0 (0) | 1 (0.3) |  | 0 | 0 | 0 (0) |
| *M. marinum* | 0 (0) | 1 (0.42) | 1 (0.3) |  | 0 | 1 (0.88) | 1 (0.67) |
| *M. monacense* | 0 (0) | 0 (0) | 0 (0) |  | 0 | 0 | 0 (0) |
| *M. mucogenicum* | 0 (0) | 0 (0) | 0 (0) |  | 0 | 0 | 0 (0) |
| *M. palustre* | 0 (0) | 0 (0) | 0 (0) |  | 0 | 0 | 0 (0) |
| *M. saskatchewanense* | 0 (0) | 0 (0) | 0 (0) |  | 0 | 0 | 0 (0) |
| *M. scrofulaceum* | 3 (3.09) | 8 (3.36) | 11 (3.28) |  | 0 | 3 (2.63) | 3 (2) |
| *M. simiae* | 1 (1.03) | 0 (0) | 1 (0.3) |  | 1 (2.78) | 0 | 1 (0.67) |
| *M. szulgai* | 0 (0) | 1 (0.42) | 1 (0.3) |  | 0 | 0 | 0 (0) |
| *Mycobacterium* spp. | 11 (11.34) | 17 (7.14) | 28 (8.36) |  | 1 (2.78) | 8 (7.02) | 9 (6) |
| Rapidly grower | 1 (1.03) | 25 (10.5) | 26 (7.76) |  | 0 | 6 (5.26) | 6 (4) |
| Mixed NTM isolation | 2 (2.06) | 8 (3.36) | 10 (2.99) |  | 14 (38.89) | 32 (28.07) | 46 (30.67) |
| Multiple site infection | - | - | - |  | 0 (0) | 11 (9.65) | 11 (7.33) |
| **Total** | **97 (100)** | **238 (100)** | **335 (100)** |  | **36 (100)** | **114 (100)** | **150 (100)** |

**Note:** NTMs were isolated from 97 pulmonary samples (36 cases) and 238 extra-pulmonary samples (114 cases). Mixed NTM refers to isolation of >1 species of NTM from the specimens, i.e. from pulmonary samples includes *M. intracellulare* and *M. avium* (1 case) and *M. massiliense* and *M. abscessus* (1 case), and from extra-pulmonary samples includes MAC and *M. intracellulare* (1 case), *M. gordonae* and *M. simiae* (2 cases), *M. fortuitum* and *M. abscessus* (1 case), *M. fortuitum* and *M. peregrinum* (3 cases) and *M. intracellulare* and *M. scrofulaceum* (1 case). MAC=*Mycobacterium avium* complex.
