# Supplementary material for: Epidemiology of and risk factors for extrapulmonary nontuberculous mycobacterial infections in Northeast Thailand
Source: PeerJ. 2018 Aug 16;6:e5479. doi: 10.7717/peerj.5479 (PMC6098943; doi:10.7717/peerj.5479)
Supplement: Supplemental Information 3 [file peerj-06-5479-s003.docx]

**Table S3** Risk factors of pulmonary NTM infection (36 cases).

| **NTM infection**  **(n=36 cases)** | **Age: mean (SD)** | **Gender (M:F)** | **BMI: mean (SD)** | **anti–IFN-γ n (%)** | **Underlying diseases** | | | | | | | | | | | | | |  |
| --- | --- | --- | --- | --- | --- | --- | --- | --- | --- | --- | --- | --- | --- | --- | --- | --- | --- | --- | --- |
|  |  |  |  |  | **HIV n (%)** | **Diabetes mellitus n (%)** | **Hyper tension n (%)** | **Thyroid n (%)** | **kidney disease n (%)** | **SLE n (%)** | **Reactive cutaneous lesions** | | | **Cancer n (%)** | **Benign tumor n (%)** | **Organ trans plantation n (%)** | **Chemo therapy n (%)** | **Others n (%)** | |
|  |  |  |  |  |  |  |  |  |  |  | **Sweet's syndrome n (%)** | **Eczema n (%)** | **Erythema nodusum n (%)** |  |  |  |  |  |  |
| *M. abscessus* (n=10) | 61.6 (19.89) | 1:1 | 20.17 (2.93) | 1 (100) | 1 (33.33) | 0 (0) | 0 (0) | 0 (0) | 1 (50) | 0 (0) | 0 (0) | 0 (0) | 0 (0) | 1 (50) | 0 (0) | 0 (0) | 0 (0) | 7 (22.58) | |
| MAC (all species) (n=5) |  |  |  |  |  |  |  |  |  |  |  |  |  |  |  |  |  |  | |
| *M. intracellulare* (n=3) | 63.67 (2.52) | 1:2 | 18.4 (3.97) | 0 (0) | 0 (0) | 0 (0) | 0 (0) | 0 (0) | 0 (0) | 0 (0) | 0 (0) | 0 (0) | 0 (0) | 0 (0) | 0 (0) | 0 (0) | 0 (0) | 3 (9.68) | |
| Unidentified MAC (n=2) | 35 (11.31) | 1:1 | 27.23 (5.64) | 0 (0) | 1 (33.33) | 0 (0) | 0 (0) | 0 (0) | 0 (0) | 0 (0) | 0 (0) | 0 (0) | 0 (0) | 0 (0) | 0 (0) | 0 (0) | 0 (0) | 2 (6.45) | |
| *M. chelonae* (n=1) | 55 (0) | 0:1 | 0 (0) | 0 (0) | 0 (0) | 0 (0) | 0 (0) | 0 (0) | 0 (0) | 0 (0) | 0 (0) | 0 (0) | 0 (0) | 0 (0) | 0 (0) | 0 (0) | 0 (0) | 0 (0) | |
| *M. fortuitum* (n=3) | 55 (16.52) | 3:0 | 19.57 (1.54) | 0 (0) | 1 (33.33) | 0 (0) | 0 (0) | 0 (0) | 0 (0) | 0 (0) | 0 (0) | 0 (0) | 0 (0) | 0 (0) | 0 (0) | 0 (0) | 0 (0) | 3 (9.68) | |
| *M. godone* (n=1) | 72 (0) | 0:1 | 19.93 (0) | 0 (0) | 0 (0) | 0 (0) | 0 (0) | 0 (0) | 0 (0) | 0 (0) | 0 (0) | 0 (0) | 0 (0) | 0 (0) | 0 (0) | 0 (0) | 0 (0) | 1 (3.23) | |
| *M. simiae* (n=1) | 55 (0) | 0:1 | 0 (0) | 0 (0) | 0 (0) | 0 (0) | 0 (0) | 0 (0) | 0 (0) | 0 (0) | 0 (0) | 0 (0) | 0 (0) | 0 (0) | 0 (0) | 0 (0) | 0 (0) | 1 (3.23) | |
| *Mycobacterium* spp. (n=1) | 56 (0) | 0:1 | 18.76 (0) | 0 (0) | 0 (0) | 0 (0) | 0 (0) | 0 (0) | 0 (0) | 0 (0) | 0 (0) | 0 (0) | 0 (0) | 0 (0) | 0 (0) | 0 (0) | 0 (0) | 1 (3.23) | |
| Mixed infection (n=14) | 55.43 (20.43) | 3:4 | 19.92 (3.92) | 0 (0) | 0 (0) | 1 (100) | 3 (100) | 0 (0) | 1 (50) | 1 (100) | 0 (0) | 0 (0) | 0 (0) | 1 (50) | 1 (100) | 0 (0) | 0 (0) | 13 (41.94) | |
| **Total** | **57.11 (17.94)** | **4:5** | **20.27 (3.84)** | **1 (100)** | **3 (100)** | **1 (100)** | **3 (100)** | **0 (0)** | **2 (100)** | **1 (100)** | **0 (0)** | **0 (0)** | **0 (0)** | **2 (100)** | **1 (100)** | **0 (0)** | **0 (0)** | **31 (100)** | |

NTMs were isolated from 36 patients with pulmonary infections. Mixed infection (14 cases) refers to >1 species of NTM isolated from the same specimen or multiple specimen types from an individual patient. Missing data (no relevant data specified in the medical records) for BMI (n=8 cases), anti–IFN-γ (n=35 cases,) and HIV (n=22 cases). Patients for whom tests for HIV and /or anti–IFN-γ autoantibodies were not done had presented none of the associated symptoms and/or, in the case of HIV, had no risk factors reported in their histories. We have treated these cases as negative results in the analysis. “Others” refers to asthma, chronic obstructive pulmonary disease, pneumonia, bronchiectasis, cryptococcosis, meningitis, pyelonephritis, allergy, allergic rhinitis, rheumatoid arthritis, sinusitis and liver disease. MAC=*Mycobacterium avium* complex.
