# Supplementary material for: Epidemiology of and risk factors for extrapulmonary nontuberculous mycobacterial infections in Northeast Thailand
Source: PeerJ. 2018 Aug 16;6:e5479. doi: 10.7717/peerj.5479 (PMC6098943; doi:10.7717/peerj.5479)
Supplement: Supplemental Information 4 — NTMs were isolated from 114 patients with extra-pulmonary infections. Mixed infection (32 cases) refers to >1 species of NTM isolated from the same specimen or multiple specimen types from a single individual. Multi-organ infection (11 cases) refers to isolation of the same NTM species from various organ sites from an individual patient. Missing data (no test result or record specified in the medical records) for BMI (n = 30 cases), anti–IFN-γ (n = 59 case) and HIV (n = 38 cases). Patients for whom tests for HIV and /or anti–IFN-γ autoantibodies were not done had presented none of the associated symptoms and/or, in the case of HIV, had no risk factors reported in their histories. We have treated these cases as negative results in the analysis. “Others” referred to cholestatic hepatitis, histoplasmosis, arthritis, cryptococcosis, fungal keratitis, liver disease, osteonecrosis, thalassaemia, shigellosis, melioidosis, myelodysplastic syndromes, pneumonia, cerebrovascular disease, Kaposi’s sarcoma, bronchitis, Parkinson’s disease, parasitic diseases, plane wart, Salmonella septicaemia, lymphadenopathy, necrotising fasciitis and Kimura’s disease. MAC = Mycobacterium avium complex, RGM = Rapid growing Mycobacteria. [file peerj-06-5479-s004.docx]

**Table S4** Risk factors for extrapulmonary NTM infection (114 cases).

| **NTM infection**  **(n=114 cases)** | **Age: mean (SD)** | **Gender (M:F)** | **BMI: mean (SD)** | **anti–IFN-γ n (%)** | **Underlying diseases** | | | | | | | | | | | | | |  |
| --- | --- | --- | --- | --- | --- | --- | --- | --- | --- | --- | --- | --- | --- | --- | --- | --- | --- | --- | --- |
|  |  |  |  |  | **HIV n (%)** | **Diabetes mellitus n (%)** | **Hyper tension n (%)** | **Thyroid n (%)** | **kidney disease n (%)** | **SLE n (%)** | **Reactive cutaneous lesions** | | | **Cancer n (%)** | **Benign tumor n (%)** | **Organ trans plantation n (%)** | **Chemo therapy n (%)** | **Others n (%)** | |
|  |  |  |  |  |  |  |  |  |  |  | **Sweet's syndrome n (%)** | **Eczema n (%)** | **Erythema nodusum n (%)** |  |  |  |  |  |  |
| *M. abscessus* (n=29) | 53.76 (10.08) | 14:15 | 22.38 (3.76) | 12 (22.22) | 2 (14.29) | 3 (25) | 5 (33.33) | 0 (0) | 6 (28.57) | 1 (20) | 4 (22.22) | 4 (44.44) | 0 (0) | 5 (31.25) | 1 (12.5) | 4 (50) | 1 (25) | 16 (26.67) | |
| *M.asiaticum* (n=1) | 82 (0) | 0:1 | 0 (0) | 1 (1.85) | 0 (0) | 1 (8.33) | 0 (0) | 0 (0) | 1 (4.76) | 0 (0) | 0 (0) | 0 (0) | 0 (0) | 0 (0) | 0 (0) | 0 (0) | 0 (0) | 1 (1.67) | |
| MAC (all species) (n=17) |  |  |  |  |  |  |  |  |  |  |  |  |  |  |  |  |  |  | |
| *M. avium* (n=3) | 38 (10.15) | 2:1 | 18.9 (0) | 0 (0) | 2 (14.29) | 0 (0) | 0 (0) | 0 (0) | 0 (0) | 0 (0) | 0 (0) | 1 (11.11) | 0 (0) | 0 (0) | 0 (0) | 0 (0) | 0 (0) | 2 (3.33) | |
| *M. intracellulare* (n=7) | 55.43 (15.95) | 4:3 | 21.74 (3.44) | 3 (5.56) | 0 (0) | 0 (0) | 1 (6.67) | 0 (0) | 1 (4.76) | 1 (20) | 0 (0) | 0 (0) | 0 (0) | 0 (0) | 1 (12.5) | 0 (0) | 0 (0) | 2 (3.33) | |
| Unidentified MAC (n=7) | 38 (12.22) | 7:0 | 20.97 (3.87) | 1 (1.85) | 5 (35.71) | 0 (0) | 0 (0) | 0 (0) | 0 (0) | 0 (0) | 0 (0) | 0 (0) | 0 (0) | 1 (6.25) | 0 (0) | 0 (0) | 0 (0) | 4 (6.67) | |
| *M. chelonae* (n=3) | 50 (19.97) | 2:1 | 20.75 (2.87) | 1 (1.85) | 1 (7.14) | 0 (0) | 0 (0) | 0 (0) | 1 (4.76) | 0 (0) | 1 (5.56) | 0 (0) | 0 (0) | 0 (0) | 0 (0) | 0 (0) | 0 (0) | 1 (1.67) | |
| *M. fortuitum* (n=2) | 73 (7.07) | 0:1 | 24.45 (6.29) | 0 (0) | 0 (0) | 0 (0) | 0 (0) | 0 (0) | 1 (4.76) | 0 (0) | 0 (0) | 0 (0) | 0 (0) | 0 (0) | 0 (0) | 0 (0) | 0 (0) | 2 (3.33) | |
| *M. genavense* (n=1) | 57 (0) | 0:1 | 22.22 (0) | 1 (1.85) | 0 (0) | 0 (0) | 0 (0) | 0 (0) | 0 (0) | 0 (0) | 1 (5.56) | 0 (0) | 0 (0) | 0 (0) | 0 (0) | 0 (0) | 0 (0) | 0 (0) | |
| *M. marinum* (n=1) | 44 (0) | 1:0 | 0 (0) | 0 (0) | 0 (0) | 0 (0) | 0 (0) | 0 (0) | 0 (0) | 0 (0) | 0 (0) | 0 (0) | 0 (0) | 0 (0) | 0 (0) | 0 (0) | 0 (0) | 0 (0) | |
| *M. scrofulaceum* (n=3) | 56.67 (9.45) | 1:2 | 21.62 (4.16) | 1 (1.85) | 0 (0) | 0 (0) | 0 (0) | 0 (0) | 0 (0) | 0 (0) | 0 (0) | 0 (0) | 0 (0) | 2 (12.5) | 0 (0) | 0 (0) | 0 (0) | 1 (1.67) | |
| *Mycobacterium* spp. (n=8) | 42.88 (17.11) | 1:3 | 26.5 (4.42) | 2 (3.7) | 1 (7.14) | 0 (0) | 0 (0) | 0 (0) | 1 (4.76) | 1 (20) | 2 (11.11) | 1 (11.11) | 0 (0) | 1 (6.25) | 0 (0) | 0 (0) | 0 (0) | 4 (6.67) | |
| RGM (n=6) | 54.33 (13.95) | 1:1 | 24 (6.56) | 2 (3.7) | 0 (0) | 3 (25) | 3 (20) | 0 (0) | 3 (14.29) | 0 (0) | 0 (0) | 0 (0) | 0 (0) | 0 (0) | 0 (0) | 2 (25) | 1 (25) | 3 (5) | |
| Mixed infection (n=32) | 49.47 (13.23) | 15:17 | 21.82 (4.76) | 22 (40.74) | 3 (21.43) | 5 (41.67) | 5 (33.33) | 2 (40) | 7 (33.33) | 2 (40) | 10 (55.56) | 3 (33.33) | 1 (100) | 5 (31.25) | 5 (62.5) | 1 (12.5) | 2 (50) | 19 (31.67) | |
| Multi-organ infection (n=11) | 44.18 (19.91) | 2:9 | 22.03 (3.3) | 8 (14.81) | 0 (0) | 0 (0) | 1 (6.67) | 3 (60) | 0 (0) | 0 (0) | 0 (0) | 0 (0) | 0 (0) | 2 (12.5) | 1 (12.5) | 1 (12.5) | 0 (0) | 5 (8.33) | |
| **Total** | **50.12 (14.63)** | **53:61** | **22.37 (4.22)** | **54 (100)** | **14 (100)** | **12 (100)** | **15 (100)** | **5 (100)** | **21 (100)** | **5 (100)** | **18 (100)** | **9 (100)** | **1 (100)** | **16 (100)** | **8 (100)** | **8 (100)** | **4 (100)** | **60 (100)** | |

NTMs were isolated from 114 patients with extra-pulmonary infections. Mixed infection (32 cases) refers to >1 species of NTM isolated from the same specimen or multiple specimen types from a single individual. Multi-organ infection (11 cases) refers to isolation of the same NTM species from various organ sites from an individual patient. Missing data (no test result or record specified in the medical records) for BMI (n=30 cases), anti–IFN**-**γ (n=59 case) and HIV (n=38 cases). Patients for whom tests for HIV and /or anti–IFN-γ autoantibodies were not done had presented none of the associated symptoms and/or, in the case of HIV, had no risk factors reported in their histories. We have treated these cases as negative results in the analysis. “Others” referred to cholestatic hepatitis, histoplasmosis, arthritis, cryptococcosis, fungal keratitis, liver disease, osteonecrosis, thalassaemia, shigellosis, melioidosis, myelodysplastic syndromes, pneumonia, cerebrovascular disease, Kaposi's sarcoma, bronchitis, Parkinson’s disease, parasitic diseases, plane wart, Salmonella septicaemia, lymphadenopathy, necrotising fasciitis and Kimura's disease. MAC=*Mycobacterium avium* complex, RGM= Rapid growing Mycobacteria.
