# Supplementary material for: Epidemiology of and risk factors for extrapulmonary nontuberculous mycobacterial infections in Northeast Thailand
Source: PeerJ. 2018 Aug 16;6:e5479. doi: 10.7717/peerj.5479 (PMC6098943; doi:10.7717/peerj.5479)
Supplement: Supplemental Information 5 — Mixed infection refers to >1 species of NTM isolated from the sample specimen or multiple specimens from the same NTM-infected case. Multi-organ infection refers to single NTM species isolated from multiple organ sites from an individual patient. From the total of 57 cases, 41 were defined based on NTM isolation from sterile sites only. In 16 cases, NTMs were isolated from both sterile and non-sterile specimens including 10 cases of mixed infection and 6 cases of multiple-organ infection. MAC = Mycobacterium avium complex. [file peerj-06-5479-s005.docx]

**Table S5** Details of mixed infections and multi-organ NTM infections (total = 57 cases).

|  | **NTM infections** | **No. of cases** | **Notes** |
| --- | --- | --- | --- |
| **Mixed infection (n=46)** | | | |
|  | *M. abscessus* and *M. chelonae* | 2 | 1 case (Sputum & Lymph node), 1 case (Sputum) |
|  | *M. abscessus* and *M. fortuitum* | 3 | 1 case (Sputum), 1 case (Bronchial wash), 1 case (Pericardium fluid & Pleural fluid) |
|  | *M. abscessus*, *M. gordonae* and *Mycobacterium* spp. | 1 | Sputum |
|  | *M. abscessus* and *M. intracellulare* | 2 | 1 case (Bronchial wash, Tracheal suction & Lymph node), 1 case (Sputum & Tissue) |
|  | *M. abscessus*, *M. intracellulare*, *M. chelonae* and *Mycobacterium* spp. | 1 | Sputum |
|  | *M. abscessus*, *M. intracellulare*, *M. kansasii*, M*. malmoense* and *Mycobacterium* spp. | 1 | Sputum |
|  | *M. abscessus*, *M. intracellulare* and *Mycobacterium* spp. | 2 | Sputum |
|  | *M. abscessus* and *M. massiliense* | 1 | Sputum |
|  | *M. abscessus* and RGM | 10 | 1 case (Blood, Tissue, Lymph node & Other source), 3 cases (Lymph node), 1 case (Urine & Others source), 1 case (Sputum & Other source), 1 case (Sputum), 1 case (Blood), 1 case (Tissue & Lymph node), 1 case (Lymph node & Other source), |
|  | *M. abscessus*, RGM and *M. kansasii* | 1 | Lymph node, Tissue |
|  | *M. abscessus* and *Mycobacterium* spp. | 4 | 3 cases (Lymph node), 1 case (Lymph node & Pleural fluid) |
|  | MAC and *M. abscessus* | 2 | 1 case (Blood, Lymph node & Bronchoalveolar Lavage), 1 case (Blood, Lymph node & Sputum) |
|  | MAC, *M. intracellulare* | 2 | 1 case (Bone marrow), 1 case (Sputum, Lymph node & Tissue) |
|  | MAC, *M. intracellulare* and *M. gordonae* | 1 | Sputum |
|  | *M. avium* and *M. intracellulare* | 1 | Pleural fluid |
|  | *M. chelonae* and *Mycobacterium* spp. | 1 | Sputum & Lymph node |
|  | *M. fortuitum* and *M. peregrinum* | 3 | 3 cases (Blood) |
|  | *M. fortuitum* and *Mycobacterium* spp. | 1 | Pus |
|  | *M. gordonae* and *M. simiae* | 2 | 2 cases (Bone Marrow) |
|  | *M. intracellulare*, *M. gordonae* and *M. scrofulaceum* | 1 | Sputum |
|  | *M. intracellulare* and *M. scrofulaceum* | 2 | 1 case (Blood, Bone marrow, Lymph node & Tissue), 1 case (Fluid, Nasal cavity, Cheeks & Pus from wound) |
|  | *M. intracellulare* and *M. scrofulaceum* and *Mycobacterium* spp. | 1 | Sputum |
|  | *M. szulgai* and *Mycobacterium* spp. | 1 | Lymph node & Knee fluid |
| **Multi-organ infection (n=11)** | |  |  |
|  | *M. abscessus* | 5 | 1 case (Lymph node & Axillary tissue), 1 case (Lymph node & Tonsil), 1 case (Unidentified tissue &Trachea tissue), 1 case (Neck & Tracheal suction), 1 case (Humerous tissue & Other source) |
|  | MAC | 1 | Pus from wound, Chest (pus), Shoulder, Thigh(pus), Elbow(fluid), Sputum & Synovial fluid |
|  | *M. intracellulare* | 3 | 1 case (Lymph node, Neck, Tissue, pus & arm), 1 case (Gluteal, Bone marrow & Sputum), 1 case (Fluid &Tissue) |
|  | *M. kansasii* | 1 | Blood, Pleural Fluid lymph node & sputum |
|  | *M. scrofulaceum* | 1 | Pus from wound & sputum |

Mixed infection refers to >1 species of NTM isolated from the sample specimen or multiple specimens from the same NTM-infected case. Multi-organ infection refers to single NTM species isolated from multiple organ sites from an individual patient. From the total of 57 cases, 41 were defined based on NTM isolation from sterile sites only. In 16 cases, NTMs were isolated from both sterile and non-sterile specimens including 10 cases of mixed infection and 6 cases of multiple-organ infection. MAC=*Mycobacterium avium* complex.
